# Dr. Cousins

**Published:** 1869-02

**Authors:** 


Dr. Cousins.—We learn from Dr. S. H. Sawyer, of Unionville, that Dr. Cousins, of Albia, in this State, died a few days since of Tuberculosis. The Doctor was an old citizen of Marion county, and has occupied a prominent position in the profession for many years. We sympathize with his friends and the profession in the loss they have sustained.

